# Carbon-nanoparticle-triggered acute lung inflammation and its resolution are not altered in PPARγ-defective (P465L) mice

**DOI:** 10.1186/1743-8977-8-28

**Published:** 2011-09-20

**Authors:** Alexander A Götz, Antonio Vidal-Puig, Heiko G Rödel, Martin Hrabé de Angelis, Tobias Stoeger

**Affiliations:** 1 Comprehensive Pneumology Center, Institute of Lung Biology and Disease, Helmholtz Zentrum München, German Research Center for Environmental Health, Ingolstaedter Landstrasse 1, Neuherberg/Munich, D-85764, Germany; 2 Metabolic Research Laboratories, Level 4, Institute of Metabolic Science, Box 289, NIHR Cambridge Biomedical Research Centre Addenbrooke's Hospital, University of Cambridge, Cambridge, CB2 0QQ, UK; 3 Laboratory of Experimental and Comparative Ethology, University of Paris 13, F-93430, Villetaneuse, France; 4 Institute of Experimental Genetics, Helmholtz Zentrum München, German Research Center for Environmental Health, Ingolstaedter Landstrasse 1, Neuherberg/Munich, D-85764, Germany

**Keywords:** peroxisome-proliverator activated receptor γ, carbon-nano particle, pulmonary inflammation, chronic lung disease, challenge, immune cell, broncho-alveolar lavage (BAL), inflammatory marker

## Abstract

**Background:**

The alveolar macrophage (AM) - first line of innate immune defence against pathogens and environmental irritants - constitutively expresses peroxisome-proliferator activated receptor γ (PPARγ). PPARγ ligand-induced activation keeps the AM quiescent, and thereby contributes to combat invaders and resolve inflammation by augmenting the phagocytosis of apoptotic neutrophils and inhibiting an excessive expression of inflammatory genes. Because of these presumed anti-inflammatory functions of PPARγ we tested the hypothesis, whether reduced functional receptor availability in mutant mice resulted in increased cellular and molecular inflammatory response during acute inflammation and/or in an impairment of its resolution.

**Methods:**

To address this hypothesis we examined the effects of a carbon-nanoparticle (CNP) lung challenge, as surrogate for non-infectious environmental irritants, in a murine model carrying a dominant-negative point mutation in the ligand-binding domain of PPARγ (P465L/wt). Animals were instilled intratracheally with Printex 90 CNPs and bronchoalveolar lavage (BAL) was gained 24 h or 72 h after instillation to investigate its cellular and protein composition.

**Results:**

Higher BAL cell numbers - due to higher macrophage counts - were found in mutants irrespective of treatment. Neutrophil numbers in contrast were slightly lower in mutants. Intratracheal CNP instillation resulted in a profound recruitment of inflammatory neutrophils into the alveolus, but genotype related differences at acute inflammation (24 h) and resolution (72 h) were not observed. There were no signs for increased alveolar-capillary membrane damage or necrotic cell death in mutants as determined by BAL protein and lactate-dehydrogenase content. Pro-inflammatory macrophage-derived cytokine osteopontin was higher, but galectin-3 lower in female mutants. CXCL5 and lipocalin-2 markers, attributed to epithelial cell stimulation did not differ.

**Conclusions:**

Despite general genotype-related differences, we had to reject our hypothesis of an increased CNP induced lung inflammation and an impairment of its resolution in PPARγ defective mice. Although earlier studies showed ligand-induced activation of nuclear receptor PPARγ to promote resolution of lung inflammation, its reduced activity did not provide signs of resolution impairment in the settings investigated here.

## Background

The peroxisome proliferator-activated receptor γ (PPARγ) is expressed in several organs and tissues [1-3] and is involved in the regulation of adipocyte differentiation and glucose homeostasis [4-7], being a regulator of energy homeostasis. PPARγ has been involved in lung maturation in mice [3,8] and its expression was found in immune cells, like lymphocytes, macrophages, and granulocytes, the latter mainly involved in inflammatory reactions [9,10]. PPARγ acts as a ligand-activated transcription factor [11]. Prostaglandins [8,12], but also synthetic and nonsteroidal anti-inflammatory substances [8,13] activate the receptor. PPARγ activation has been shown to exhibit anti-inflammatory potential by inhibiting the activity of pro-inflammatory transcription factors such as e.g. the activator protein 1 (AP-1), signal transducer and activators of transcription (STATs), or the Nuclear factor kappa B (NF-κB), as shown in murine primary peritoneal macrophages [14-16].

In particular alveolar macrophages (AM) have increased levels of PPARγ [9] and are constantly bathed in lipid-rich surfactant [17] that consists of potential receptor activating ligands, or at least precursors of ligands [14,18,19]. This coexistence of high levels of PPARγ in an environment rich in lipophilic ligands is an important finding, since: i) resident AMs in the alveolus represent the first line of innate immune defence in the respiratory tract and ii) AM orchestrate inflammatory responses by recognizing tissue damage, promoting neutrophil recruitment for appropriate pathogen defence and finally leading to resolution of inflammation [20]. This indispensable role in lung homeostasis makes the AM a promising target for the treatment of inflammatory lung diseases. In fact murine studies have revealed AM function requires upregulation of the expression of CD36, a PPARγ target. CD36 is a cell surface scavenger receptor and a key factor promoting phagocytosis of apoptotic neutrophils, lipids and unopsonized materials [18]. Similarly, an increase in Fcγ receptor mediated phagocytosis of opsonized materials [21] seems to require PPARγ activation. This AM cell-mediated effector promoting resolution of inflammation depends on the PPARγ-induced molecular anti-inflammatory properties [22] as well as by factors of different lung structural cell types, thereby down-regulating pro-inflammatory mediators [10] like TNFα, neutrophil and monocyte-macrophage chemotactic factors IL-8, MCP-1, pro-oxidant enzyme iNOS, and MMP9 [23-25] while up-regulating expression of anti-inflammatory proteins like IL-10 (reviewed in [9]). These results suggest a potential therapeutic application of PPARγ activation to resolve lung inflammatory disorders. This is particularly relevant since AM play a critical role in pathogenesis of asthma, chronic obstructive pulmonary disease (COPD), lung fibrosis (IPF) and lung sarcoidosis (for review see [9]). Moreover PPARγ binding to the respective response elements in AMs is markedly reduced in chronic inflammatory pulmonary sarcoidosis and obstructive diseases [26,27]. This suggests that the alveolar microenvironment might be immuno-suppressive in the absence of a specific stimulus [28], keeping the AM in a quiescent mode possibly supported by PPARγ function.

PPARγ knockout models have already revealed developmental airspace enlargement, and greater smoke-induced emphysema, with increased AM numbers [3,8]. In agreement with this beneficial effects of ligand-induced PPARγ activation in the lung [8,29] have been suggested, as indicated by the attenuation of pro-inflammatory cytokine release from activated AMs, eosinophils and type2 epithelial cells [29], and reduced smoke-induced epithelial mucin production [30]. Improved pathophysiological states in models for asthma, COPD, IPF, and acute lung injury have also been found [29,31-33]. In contrast, PPARγ deficiency or lack of receptor activation in macrophages resulted in increased atherosclerosis [34] and reduced CD36 expression [18,35]. Take together all together, these findings highlight PPARγ as a promising target for the treatment of many inflammatory pathologies by promoting resolution of inflammation [18].

According to these anti-inflammatory effects in the lung and the fact that unresolved pulmonary inflammation may lead to chronic disease states, we tested the hypothesis that a diminished PPARγ function may result in an increased cellular and molecular inflammatory response, during acute inflammation and impaired resolution. With regard to an inflammatory stimulation of the lungs by particulate matter, so far PPARγ function has only been associated with exposure to cigarette smoke but not with environmental particles such as combustion derived nanoparticles. To address this hypothesis we investigated mice (C57BL/6J) carrying a dominant-negative point mutation (P465L) in the ligand-binding domain of the PPARγ receptor - a targeted mutation, equivalent to a rare mutation in humans (P467L) [5,36-38]. Whereas human carriers of the mutation suffer from lipodystrophy, extreme insulin resistance, as well as hypertension, fatty liver, and lower adiponectin levels in circulation, humans with the homozygous for P465L die in utero. Mice with the same mutation developed apparently morphologically normal total amounts of adipose tissue - although displaying higher extra-abdominal fat mass - and were insulin sensitive [6,7]. However, these animals recapitulated the human phenotype once challenged with positive energy balance [7]. We favoured to use P465L/wt mutant mice over the more severely compromised PPARγ knock-out mice since it more reliably resembles the situation in chronic inflammatory lung diseases as described, in alveolar macrophages - like in asthma [39], pulmonary sarcoidosis [26,27] and COPD [9] - or in epithelial cells like in cystic fibrosis [40], where PPARγ activation was found to be reduced, but not absent. Our rational was that if PPARγ contributes to an anti-inflammatory macrophage state and/or is involved in the resolution of inflammation, then PPARγ defective mice should show impaired resolution of particle induced lung inflammation, a model clearly involving alveolar macrophage function [41,42].

To our knowledge, apart from cigarette smoke, yet no one has investigated PPARγ related effects in the context of particle related lung inflammation. Exposure to Printex 90 was primarily chosen as a surrogate for urban air pollution by combustion derived nanoparticles. However since in addition to its generation by combustion processes like from diesel engines, carbon black is a constituent of lots of products of modern societies, like inks and paints, rubber and plastic, and thus progressively becoming a more relevant anthropogenic source of ambient and indoor particulate matter. In fact more than 10 million tones are produced every year [43]. But regardless of CNP ancestry, whether airborne, combustion derived or engineered, this sub-100 nm scaled particle class has gained toxicological interest due to their small dimensions, large surface area and high deposition efficiency in the lung being considered an important driver of adverse health effects linked to respiratory toxicity [44,45]. It is widely accepted that particulate air pollution contributes to the adverse health effects in humans and that patients with metabolic syndrome (obesity, hypertension and diabetes mellitus) may be a more susceptible population. Thus the identification of underlying pathways linking the inflammatory responses induced by particle related health effects and susceptibility to metabolic diseases are of prime importance. In this respect we speculate that PPARγ might be one of the connections linking the regulation of lipid metabolism with alveolar inflammation.

In summary our aim was on to contribute to the understanding of the pathogenic role of PPARγ biology during pulmonary inflammation caused by non-infectious respirable stimuli as represented by carbonaceous particulate matter. We wanted to clarify whether the reduced availability of functional PPARγ in (P465L/wt) mutant mice increased the susceptibility towards acute inflammation and failed resolution in response to CNP-stimulus in comparison to PPARγ wild-type mice (wt/wt). Experiments were performed in adult, 12-14 weeks old, PPARγ wild-type (wt/wt) and P465L/wt mutant mice of both genders to account for sex-specific hormone levels [46,47]. Animals were challenged using physically and chemically well characterized CNPs of moderate toxicity as described earlier [41].

## Results

### Bronchoalveolar Lavage (BAL) cell analysis

**Figure 1:**
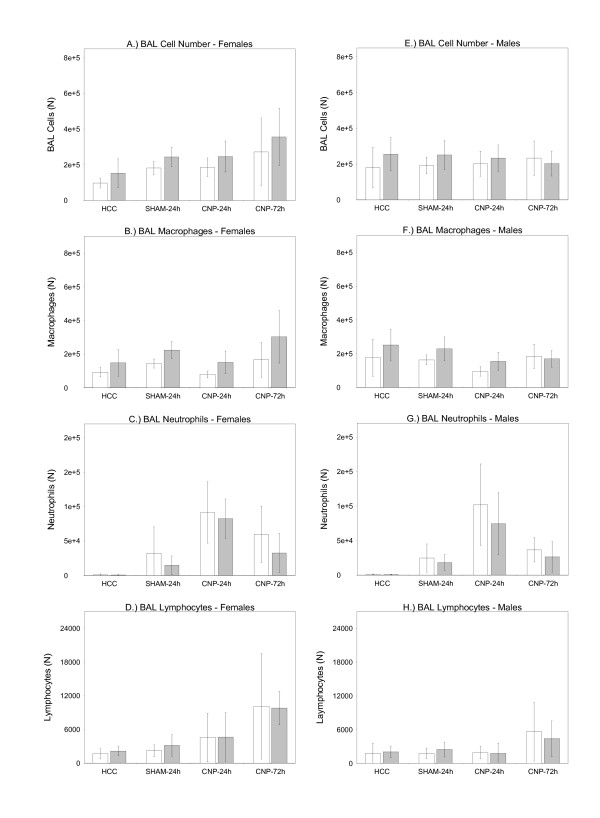
**Bronchoalveolar lavage (BAL) cell differentials**. BAL cells of female (A.-D.) and male (E.-H) PPARγ wild-type (wt/wt) (white bars) and PPARγ mutant mice (P465L/wt) (grey bars). Untreated home cage controls (HCC); water-instilled animals at 24 h time point (SHAM-24 h); particle-instilled mice at 24 h (CNP-24 h) and 72 h time point (CNP-72 h). For sample size, please see Table1. Statistics: General Linear Model (GLM): BAL Cell Number: genotype: F/W = 11.045, df = 1,**P = 0.001; treatment: F/W = 12.254, df = 3, ***P < 0.001; sex: ***P < 0.001; treatment × sex: F/W = 6.449, df = 3, ***P < 0.001; BAL Macrophages: genotype: F/W = 29.434, df = 1, ***P < 0.001; treatment: F/W = 9.767, df = 3, ***P < 0.001; sex: F/W = 14.869, df = 1, ***P < 0.001; treatment × sex: F/W = 4.697, df = 3, **P = 0.0039; BAL Neutrophils: genotype: F/W = 7.274, df = 1, **P = 0.008; treatment: F/W = 103.631, df = 3, ***P < 0.001; sex: F/W = 0.892, df = 1, P = 0.347; BAL Lymphocytes: genotype: F/W = 0.352, df = 1, P = 0.5543; treatment: F/W = 17.437, df = 3, ***P < 0.001; sex: F/W = 0.059, df = 1, P = 0.810; treatment × sex: F/W = 2.944, *P = 0.036;

Bronchoalveolar lavage (BAL) volumes obtained from age- and body mass-matched PPARγ wild-type (wt/wt) and PPARγ mutant mice (P465L/wt) did not reveal significant differences between groups allowing an adequate comparison of BAL cell differentials between groups. Cytospin analysis showed significant differences in total BAL cell numbers between genotypes. This was observed in both sexes, being generally slightly higher in the mutant group irrespectively of the treatment (Figure 1A and 1E). This effect was due to higher macrophage counts associated with the mutant genotype (Figure 1B and 1F). In contrast the BAL neutrophil pool was lightly lower in mutants (Figure 1C and 1G). Lymphocyte numbers did not differ between genotypes in both sexes (Figure 1D and 1H). Significant interaction terms (treatment × sex) indicated some sex-specific differences. Compared to males, females displayed lower total BAL cell numbers under control conditions but higher numbers at the 72 h time point. This difference was mainly reflected by initially (HCC condition) lower macrophage numbers (Figure 1B and 1F, Additional File 1, Figure s4 B) and higher numbers of macrophages at the 72 h time point (Figure 1D and 1H, Additional File 1, Figure s4). In male mice total BAL cell numbers where constant and not affected by the treatment (Figure 1A and 1E; Additional File 1, Figure s4 A). No sex-specific effect of treatment was observed for BAL neutrophil numbers (Figure 1C and 1G, Additional File 1, Figure s4 C), being considered the most significant read out for inflammation.

Especially the absence of neutrophils in all HCC groups (Figure 1C and 1G) was evidencing that there was no pro-inflammatory condition, in absence of a treatment related stimulus. Twenty-four hours after particle instillation (CNP-24 h), a significant influx of neutrophil granulocytes into the alveolar lumen was observed, indicating acute lung inflammation in both, wild-type and mutant animals (Figure 1C and 1G). However, particle instillation did not cause significant genotype-related differences in the magnitude of neutrophil recruitment into alveolar lumen (Figure 1C and 1G). Seventy-two hours after particle instillation (CNP-72 h) neutrophil numbers were significantly lower in comparison to the time point of acute lung inflammation (CNP-24 h), indicating similar degree of resolution of inflammation on a cellular level in both genotypes and sexes (Figure 1C and 1G).

### BAL: Protein and Lactate Dehydrogenase (LDH)

Consistent with the absence of any difference in the cellular component of the inflammation we did not observe differences in the alveolar-capillary barrier function and found no indications of increased lung injury in the mutant mice, as usually indicated by increased BAL protein. Total BAL protein content did not differ between PPARγ (wt/wt) and PPARγ (P465L/wt) mice of both sexes under untreated HCC, SHAM, and CNP conditions after 24 h and 72 h (Additional File 1, Figure s1 A and C), respectively. This agreed with the fact that concentrations of the intracellular enzyme LDH in BAL supernatant were not different between HCC groups. Also no differences were observed in LDH levels 24 hours or 72 hours after particle instillation between genotypes (Additional File 1, Figure s1 B and D). All together, this shows lack of differences in cell membrane damage and necrotic cell death in BAL cells between genotypes.

### BAL Inflammatory marker (ELISA)

**Figure 2:**
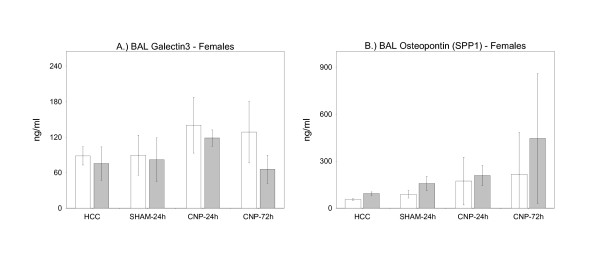
**Markers for Alveolar Macrophages**. BAL cytokine concentrations of galectin-3 (A) and osteopontin (SPP1) (B) in female PPARγ wild-type (wt/wt) (white bars) and PPARγ mutant mice (P465L/wt) (grey bars) - markers mainly derived from alveolar macrophages and known to be associated with carbon-nanoparticle-induced pulmonary inflammation. HCC: untreated home cage controls; SHAM-24 h: water-instilled animals at 24 h time point; CNP-24 h: particle-instilled mice at 24 h time point; CNP-72 h: particle-instilled mice at 72 h time point. For sample size, please see Table1. Statistics: General Linear Model (GLM): Galectin-3: genotype: F/W = 8.194, df = 1, **P = 0.006; treatment: F/W = 6.095, df = 3,**P = 0.001; SPP1: genotype: F/W = 19.786, df = 1, ***P < 0.001; treatment: F/W = 15.921, df = 3, ***P < 0.001;

**Figure 3:**
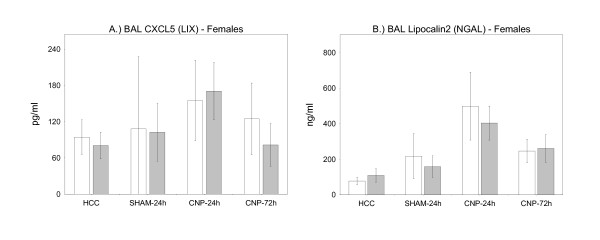
**Markers for Epithelial Cells**. BAL cytokine concentrations of CXCL5 (A), and lipocalin-2 (B) in female PPARγ wild-type (wt/wt) (white bars) and PPARγ mutant mice (P465L/wt) (grey bars) - markers mainly derived from lung epithelium, and known to be associated with carbon-nanoparticle-induced pulmonary inflammation. HCC: untreated home cage controls; SHAM-24 h: water-instilled animals at 24 h time point; CNP-24 h: particle-instilled mice at 24 h time point; CNP-72 h: particle-instilled mice at 72 h time point. For sample size, please see Table1. Statistics: General Linear Model (GLM): CXCL5: genotype: F/W = 0.205, df = 1, **P = 0.6524; treatment: F/W = 5.334, df = 3, **P = 0.003; lipocalin-2: genotype: F/W = 0.007, df = 1, P < 0.9348; treatment: F/W = 56.810, df = 3, ***P < 0.001;

Given that the cellular extent of inflammation was not different between genotypes we next searched for molecular differences in BAL inflammatory markers. We selected four pro-inflammatory proteins known to be induced by carbon-nanoparticle treatment as shown before [42,48] or known for their inflammatory/neutrophil recruiting properties. To be able to localize the response of particular cell populations we measured galectin-3 (GAL3) and osteopontin (SPP1), as predominantly alveolar macrophage derived cytokines (Figure 2A and 2B). To determine the inflammatory status of the epithelium in response to CNP instillation we investigated the BAL concentrations of anti-bacterial lipocalin-2 (LCN2/NAGL) and neutrophil recruiting cytokine CXCL5 (Figure 3A and 3B). This determination was performed in female mice only.

Analysis of alveolar macrophage derived Gal3 concentrations in BAL fluid of female PPARγ (P465L/wt) mutant mice revealed lower levels in comparison to PPARγ wild-type females (wt/wt) (GLM) under all treatment conditions (Figure 2A). In contrast, the opposite was observed for SPP1, its concentration being higher for all treatment conditions in PPARγ (P465L/wt) mutant mice in comparison with wild-type (Figure 2B). Concerning epithelial derived BAL CXCL5 and BAL lipocalin-2 no difference between genotypes was observed under whatever condition tested (Figure 3A and 3B). Values for BAL lipocalin-2 were markedly induced by particle treatment in both genotypes as indicated at 24 h time point, and were declining at 72 h time point.

### Haematological Analysis - Systemic Activation of Blood Leukocytes

Since no differences were found dependent on genotypes, gender (HCC) and treatments (SHAM; CNP-24 h; CNP-72 h) as far as BAL cell populations were concerned, we next set to investigate whether PPARγ mutations may affect the recruitment of immuno-competent leukocytes into the blood stream. Blood cell analysis did not reveal any difference at all between wild type (wt/wt) and PPARγ (P465L/wt) in both sexes, neither for total white blood cells (WBC), nor there was a difference in leukocyte subpopulations; lymphocyte, monocyte and granulocyte numbers (neutrophils, eosinophils, and basophils) (Additional File 1, Figure s2 and s3).

## Discussion

The point mutation P467L in human receptor PPARγ has been shown to be associated with adverse effects for human health and well-being, resulting in lipodystrophy, severe insulin resistance, fatty liver, hypertension, and lowered adiponectin levels in circulation [5]. In regard to this specific human situation, mice carrying a targeted point mutation in the ligand-binding domain of PPARγ (P465L), being the equivalent mutation to human P467L, were generated as an animal model, which partially confirmed the effects described in humans particularly when confronted to extreme metabolic challenges [6,7]. We used sex-, age-, and body mass-matched PPARγ mutant mice (P465L/wt) to investigate the receptor role in a particle-induced model of aseptic acute lung inflammation.

Here we show PPARγ genotype-related differences in total BAL cell numbers, with increased macrophages and reduced neutrophil counts in mutant mice. In addition, our BAL data may also indicate a pro-inflammatory shift of the M1/M2 balance of alveolar macrophages, since (i) generally higher BAL osteopontin values in mutant mice point towards a more pro-inflammatory, M1 polarized macrophage condition, and (ii) lower galectin-3 values - a marker for alternative macrophage activation - in turn indicate reduced M2 polarization. However although alternative macrophage activation is regarded as a PPARγ driven process, relevant for the resolution of inflammation, our data can not support the impact of PPARγ signalling on particle elicited lung inflammation. Our study in fact rather demonstrates that an insult with carbon-nanoparticle (CNP) challenge, administered by intratracheal instillation of Printex 90 particles to the lungs of mutant P465L/wt and wild-type mice (wt/wt) produces a similar extend of inflammatory cell recruitment during acute inflammation and resolution. That implies that the course of inflammation assessed in our lung inflammation model was not affected at cellular level by the suspected macrophage unbalance in P465L/wt mice.

Though the inflammatory reaction provoked by CNPs was mild as compared with experimental endotoxin models for instance, the response to CNP still might have been robust enough to overwhelm PPARγ pathways, and thus mask P465L/wt impairments. We have chosen an intratracheally delivered dose of 20 μg CNP, which as already previously described [41,42], resulted in marked recruitment of inflammatory cells into the alveolar lumen, without provoking significant epithelial injury. Accordingly BAL protein and BAL lactate-dehydrogenase (LDH) levels, indicators of acute lung injury and cell necrosis, did not show biologically relevant increases. The dose of 20 μg carbon particles used here represents a surface area dose of 54 cm^2 ^per mouse, an area previously related to the surface burden affected within months of people living in high polluted areas [41]. The time points of 24 h acute response phase and 72 h resolution phase are well suited to investigate the proposed hypothesis, since our results are in line with an earlier study using the same challenge design (same stimulus and dose), showing most of inflammatory neutrophil clearance in BAL fluid 72 h after challenge [42].

We can speculate that the P465L related disturbance might be limited to the macrophages, and not directly involve the epithelia compartment. P465L/wt conditions seem not effective to exacerbate/prevent the initiation or resolution of a moderate but robust, aseptic, and neutrophilic inflammation. Accordingly the epithelial-derived inflammatory marker proteins CXCL5 and lipocalin-2 did not differ between genotypes at any time point. Blood leukocyte numbers where also not affected by genotype, and did not reveal any signs for systemic inflammation upon CNP treatment. The lack of genotype related blood cell differences contrasts with the observed P465L related differences in BAL cell numbers/BAL macrophages and points towards the predominant importance of PPARγ in the alveolar region, without exhibiting systemic effects.

The absence of genotype-related differences in the cellular CNP-driven acute lung inflammation and its resolution may also be based on the possibility that P465L heterozygous mutant mice have been able to activate their mutant receptor, a possibility that may occur if high concentrations of the ligands are available. In fact it was previously shown for the respective human mutation, that increased ligand-concentrations are able to rescue the partial receptor deficit [49]. High ligand-concentrations are well conceivable for the lipid rich alveolar lining fluid presenting the direct environment of alveolar macrophages. Under this assumption future investigation would have to use a functional null of PPARγ in alveolar macrophages.

Compensation at the genomic level by an upregulation of wt-PPARγ expression in P465L/wt macrophages is not likely, since the PPARγ expression level in mutant BAL macrophages is very similar to that of wt mice (120 ± 10% of wt).

The conserved pattern of co-activator molecules used for the function of different PPARs would have been expected to contribute to a more pro-inflammatory condition in the alveolar compartment, but basal BAL levels of classically pro-inflammatory cytokines such like TNFα revealed unchanged in wt versus mutant mice (data not shown). In this context the lack of a pro-inflammatory status in blood system as well as in the alveolar compartment shows that the organism in whole can cope with the challenge even under the mutant PPARγ condition. We regard a lack of an increased pro-inflammatory situation in mutants to be related to the lipid-rich environment of the macrophage within the alveolar lining fluid, which may have compensated for a loss of receptor functionality [49].

## Conclusions

Our data contribute to the understanding of PPARγ receptor relevance in the context of alveolar macrophage biology during lung inflammation or particular resolution. In contrast to the by Asada 2004 suggested pro-resolving activity of PPARγ [18] during clearance of apoptotic neutrophils, no changes were found related to the function of this specific dominant-negative PPARγ point mutation. In order to further address and clarify the receptor's specific role in the AM-mediated resolution of pulmonary inflammation and its possible as well as suggested role in the transition towards chronic lung disease, we emphasize the need for further investigations, particularly by using macrophage specific PPARγ knock-out models.

## Methods

### Animal Generation and Genotyping

P465L/wt mutant mice were generated and genotyped as described earlier in [7,50]. P465L/wt mice where obtained from the University of Cambridge (UK) on a mixed C57BL/6-129/SvJ background and backcrossed for 9 generations to C57BL/6J for isogenicity.

### Particle Challenge Design and Group Setup

Animals were either instilled with aqueous suspension (zeta potential: 33 mV; agglomerate diameter in suspension: 0.17 μm) of Printex90 carbon-nanoparticles (CNP), a commercially available pigment black from Degussa (Frankfurt, Germany), (diameter [nm]: 14; organic content [%]: 1; surface area [m^2^/g]: 272); as described earlier in [42]), or pyrogene-free distilled water (SHAM exposed) respectively or were left undisturbed and served as controls (Home Cage Control; HCC). For details on group setup and sample size, see table 1;

**Table 1:** Group Setup and treatment

Group	Home Cage Control (HCC)	H20 - 24 h (SHAM)	Printex90 - 24 h (CNP-24 h)	Printex90 - 72 h (CNP-72 h)
Male PPARγ +/+	7	6	7	6
Male PPARγ P465L/wt	9	8	11	10
Female PPARγ +/+	8	6	7	7
Female PPARγ P465L/wt	9	9	8	6

Group Setup and treatment of investigated male and female wild-type (wt/wt) and PPARγ mutant mice (P465L/wt). Numbers of animals investigated per group are provided.

Prior to instillation, mice were anesthetized by intraperitoneal injection of a mixture of Medetomidin (0.5 mg/kg body mass), Midazolam (5.0 mg/kg body mass) and Fentanyl (0.05 mg/kg body mass). The animals were then intubated by a nonsurgical technique [51]. Using a cannula inserted 10 mm into the trachea, a suspension containing 20 μg CNPs, respectively, in 50 μl pyrogene-free distilled water was instilled, followed by 100 μl air; the suspension of poorly soluble CNPs was sonicated on ice for 1 min prior to instillation, using a SonoPlus HD70 (Bachofer, Berlin, Germany) at a moderate energy of 20 W. SHAM animals were instilled 50 μl pyrogene-free distilled water only [41]. After instillation animals were antagonized by subcutaneous injection of a mixture of Atipamezol (2.5 mg/kg body mass), Flumazenil (0.5 mg/kg body mass) and Naloxon (1.2 mg/kg body mass) to guarantee their awakening and well-being. Animals were treated humanely and with regard for alleviation of suffering; experimental protocols were reviewed and approved by the Bavarian Animal Research Authority.

### Blood, Serum, and Bronchoalveolar Lavage (BAL) sampling

Twenty-four hours or seventy-two hours after instillation, mice were anesthetized by intraperitoneal injection of a mixture of xylazine (4.1 mg/kg body weight) and ketamine (188.3 mg/kg body weight) and killed by exsanguination. Therefore blood was drawn from the retroorbital plexus by a capillary and collected a.) in EDTA covered tubes (*Sarstedt*) for haematological analysis (ADVIA Hematology Systems (Bayer Diagnostics) and b.) non EDTA-covered tubes to gain blood serum. Subsequently BAL was performed by cannulating the trachea and infusing the lungs 4 times with 1.0 ml PBS without calcium and magnesium, in adaptation as described previously [41]. The BAL fluids from lavages 1 and 2 and from lavages 3 and 4 were pooled and centrifuged (425 *g*, 20 min at room temperature). The cell-free supernatant from lavages 1 and 2 were used for biochemical measurements such as lactate dehydrogenase (LDH), total protein, and cytokine concentrations. The cell pellet was resuspended in 1 ml RPMI 1640 medium (BioChrome, Berlin, Germany) and supplemented with 10% fetal calf serum (Seromed, Berlin, Germany); the number of living cells was determined by the trypan blue exclusion method. We performed cell differentials on the cytocentrifuge preparations (May-Grünwald- Giemsa staining; 2 × 200 cells counted) and the number of polymorphonuclear leukocytes (PMNs) was used as a marker of inflammation.

### BAL: Total Protein Content and Lactate Dehydrogenase (LDH) Assay

Total BAL protein content was determined spectrophotometrically with an ELISA reader (Labsystems iEMSReader MF, Helsinki, Finland) at 620 nm, applying the Bio-Rad Protein Assay Dye Reagent (no. 500-0006; BioRad, Munich, Germany), as a potential biological marker for pulmonary capillary leakage and lung injury [52]. 5 μl BAL fluid/mouse was used for analysis.

For detection of the cytosolic enzyme lactate dehydrogenase (LDH) (U/ml), characteristic for membrane damaging effects, the Cytotoxicity Detection Kit (Roche Diagnostics, Germany) was used according to the manufacturer's instructions. LDH concentration in the BAL fluid (30 μl) was spectrophotometrically determined with an ELISA reader (Labsystems iEMS Reader MF, Helsinki, Finland) at a wavelength of 492 nm.

### BAL Cytokine Detection (ELISA)

The characteristic carbon-nanoparticle (CNP) induced alveolar macrophage inflammatory markers osteopontin (SPP1) (mouse Osteopontin; R&D Duo Sets; Catalog Number: DY441) and galectin-3 (mouse Galectin-3; R&D Duo Sets; Catalog Number: DY1197) [48], as well as the known lung mainly epithelial derived inflammatory markers LIX (CXCL5) (mouse LIX; R&D Duo Sets; Catalog Number: DY443), and lipocalin-2 (NGAL), (mouse Lipocalin-2/NGAL; R&D Duo Sets; Catalog Number: DY1857) [42,48] were assayed from BAL samples using the respective ELISA kit. One hundred μl of appropriate BAL fluid dilutions were used. Dilutions were: SPP1: 1:100; galectin-3: 1:100; lipocalin-2: 1:400; LIX: undiluted.

### Statistics

We tested the effects of the factors genotype (2 levels: wild-type (wt/wt) and mutant (P465L/wt), sex (2 levels: male, female) and treatment (4 levels: untreated home cage control (HCC), water-instilled SHAM group at 24 hours (SHAM), carbon-nanoparticle exposure at time point 24 hours (CNP-24 h), carbon-nanoparticle exposure at time point 72 hours (CNP-72 h) on different response variables by the use of a general linear model design (GLM).

We included the 2-way interaction terms of the factors (genotype × treatment, genotype × sex, sex × treatment), in order to test whether the treatment showed differential effects in relation to the different genotypes and sexes. If not statistically significant, the interaction term was reduced and the model was re-calculated. In none of the models investigated the interaction term genotype × treatment was significant (P > 0.10). Response variables, which deviated from the normal distribution, were log-, or square-root-transformed. Normality of the model residuals was checked visually by normal probability plots and with the Shapiro-Wilk test, and we assured the homogeneity of variances and goodness of fit by plotting residuals versus fitted values and by the Levene test. In case of significant interaction terms, post-hoc comparisons were conducted with the Tukey test. All statistical analyses were done using the software SPSS 14.0 (SPSS Inc., Chicago, IL)

Significant P-values by GLM testing are provided in the figure legends by asterisks (*P < 0.050; **P < 0.010; ***P < 0.001). All data are expressed as mean ± SEM.

## Competing interests

The authors declare that they have no competing interests.

## Authors' contributions

AG and TS conceived and designed the experiments. AG performed the experiments and AG and HGR analyzed the data. AG, TS, HGR, MHA, and AVP wrote the manuscript. All authors read and approved the final manuscript.

## Supplementary Material

Additional file 1 **Additional Figures s1-s4 containing data of the Bronchoalveolar Lavage (BAL) protein and Lactate Dehydrogenase (LDH) content, blood cell differentials and showing sex-specific effects of treatment on BAL cell differentials**. Click here for file
