# Supplementary material for: Carbon-nanoparticle-triggered acute lung inflammation and its resolution are not altered in PPARγ-defective (P465L) mice
Source: Part Fibre Toxicol. 2011 Sep 20;8:28. doi: 10.1186/1743-8977-8-28 (PMC3197489; doi:10.1186/1743-8977-8-28)
Supplement: Additional file 1 — Additional Figures s1-s4 containing data of the Bronchoalveolar Lavage (BAL) protein and Lactate Dehydrogenase (LDH) content, blood cell differentials and showing sex-specific effects of treatment on BAL cell differentials. [file 1743-8977-8-28-S1.PDF]

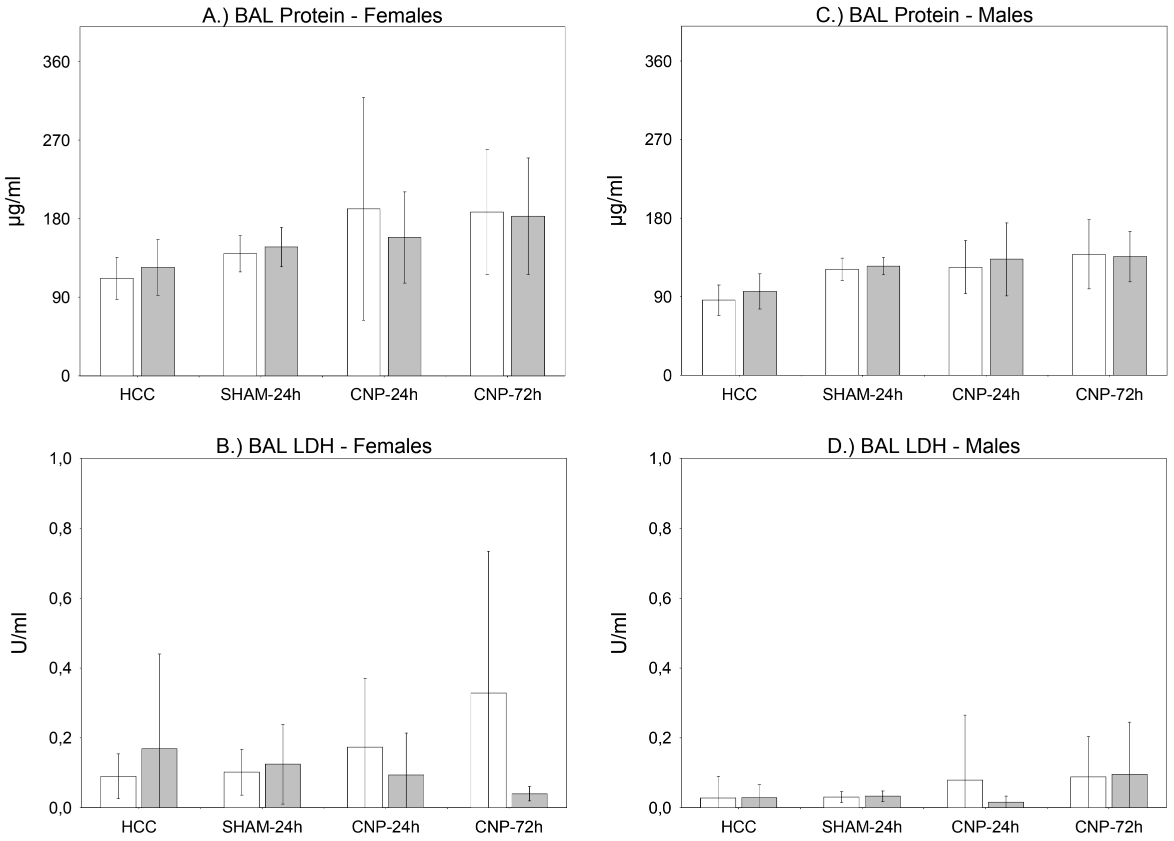

Additional Figure s1: BAL protein (A and C) and BAL Lactate Dehydrogenase (LDH) content (B and D) of female and male Pparγ wild-type (wt/wt) (white bars) and Pparγ mutant mice (P465L/wt) (grey bars). HCC: untreated home cage controls; SHAM-24h: water-instilled animals at 24h time point; CNP-24h: particle-instilled mice at 24h time point; CNP-72h: particle-instilled mice at 72h time point. For sample size, please see Table1. *Statistics: General Linear Model (GLM):*

*BAL Protein: genotype: F/W=0.328, df=1, P=0.568; treatment: F/W=14.586, df=3, ***P<0.001; sex: F/W=24.048, df=1, ***P<0.001; BAL LDH: genotype: F/W=0.745, df=1, 0.390; treatment: F/W=1.479, df=3, 0.224; sex: F/W=3.905, df=1, P=0.050;*

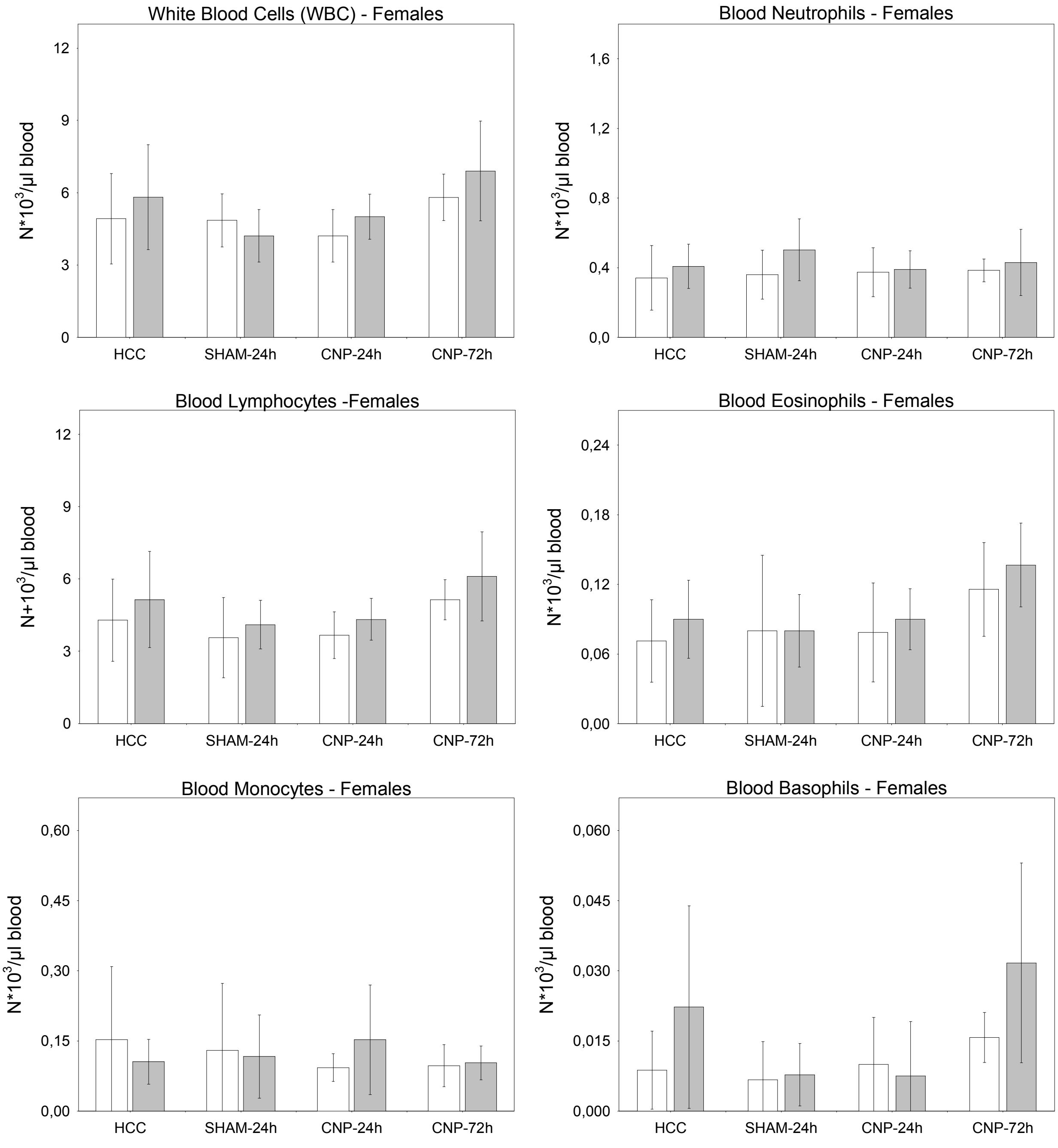

Additional Figure s2: White blood cells and leukocyte subsets of female PPARγ wild-type (wt/wt) (white bars) and PPARγ mutant mice (P465L/wt) (grey bars).HCC: untreated home cage controls; SHAM-24h: water-instilled animals at 24h time point; CNP-24h: particle-instilled mice at 24h time point; CNP-72h: particle-instilled mice at 72h time point. For sample size, please see Table1. *Statistics: General Linear Model (GLM):*

White Blood Cells: genotype: F/W=3.353, df=1, P=0.070; treatment: F/W=9.627, df=3, ***P<0.001; sex: F/W=0.178, df=1, P=0.674; Blood Lymphocytes: genotype: F/W=3.295, df=1, P=0.072; treatment: F/W=12.268, df=3, ***P<0.001; sex: F/W=1.314, df=1, P=0.254; Blood

Monocytes: genotype: F/W=0.007, df=1, P=0.934; treatment: F/W=0.235, df=3, P=0.872; sex: F/W=16.530, df=1, $***P<0.001$; Blood Neutrophils: genotype: F/W=2.803, df=1, P=0.097; treatment: F/W=3.759, df=*P=0.013; sex: F/W=11.071, df=1, **P=0.001; Blood Eosinophils: genotype: F/W=0.155, df=1, P=0.694; treatment: F/W=10.871, df=3, $***P<0.001$; sex: F/W=1.985, df=1, P=0.162; Blood Basophils: genotype: F/W=1.597, df=1, P=0.209; treatment: F/W=8.457, df=3, $***P<0.001$; sex: F/W=1.447, df=1, P=0.232;

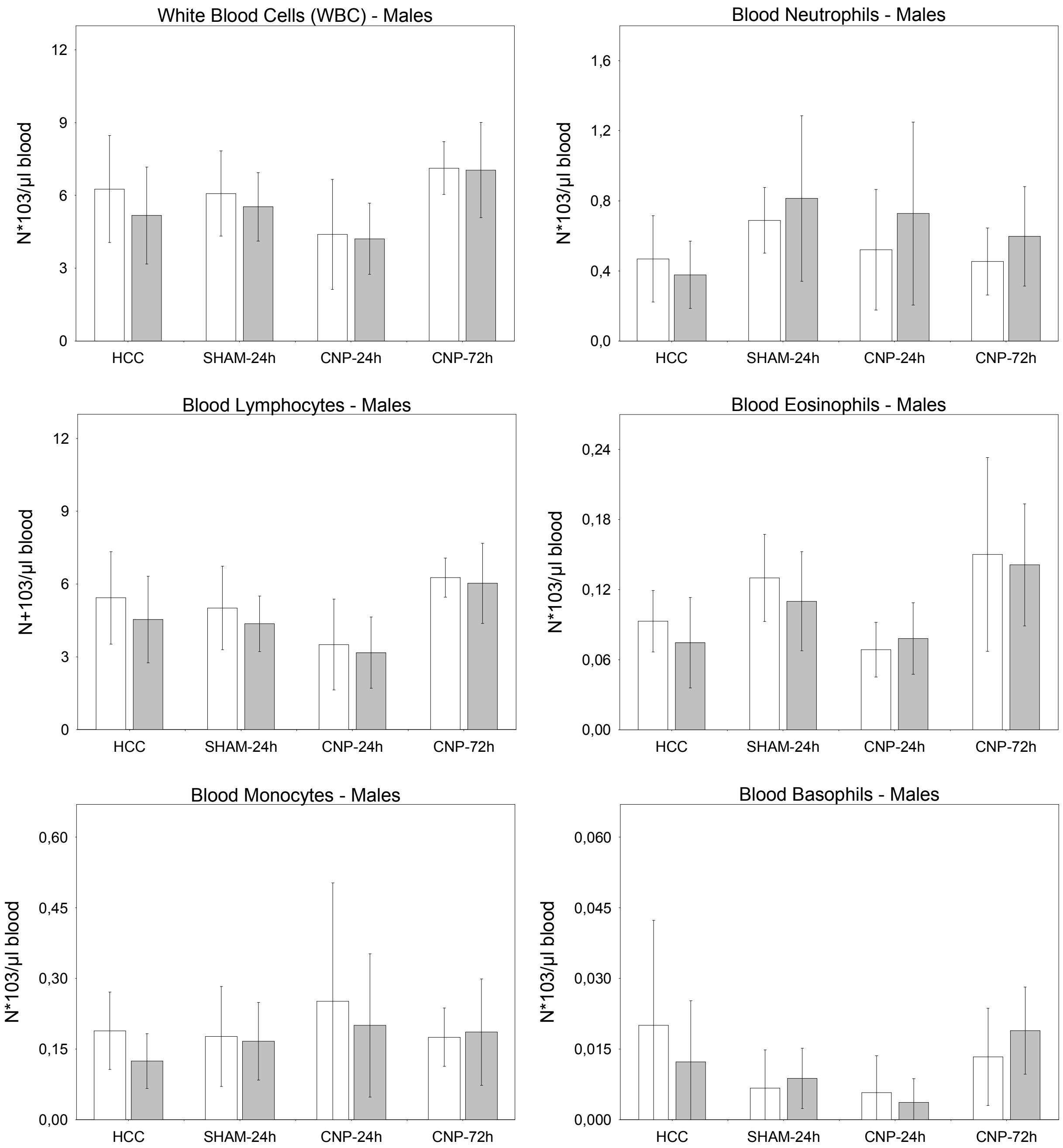

Additional Figure s3: White blood cells and leukocyte subsets of female PPARγ wild-type (wt/wt) (white bars) and PPARγ mutant mice (P465L/wt) (grey bars).HCC: untreated home cage controls; SHAM-24h: water-instilled animals at 24h time point; CNP-24h: particle-instilled mice at 24h time point; CNP-72h: particle-instilled mice at 72h time point. For sample size, please see Table1.

Statistics: General Linear Model (GLM):

White Blood Cells: genotype: F/W=3.353, df=1, P=0.070; treatment: F/W=9.627, df=3, ***P<0.001; sex: F/W=0.178, df=1, P=0.674; Blood Lymphocytes: genotype: F/W=3.295, df=1, P=0.072; treatment: F/W=12.268, df=3, ***P<0.001; sex: F/W=1.314, df=1, P=0.254; Blood

Monocytes: genotype: F/W=0.007, df=1, P=0.934; treatment: F/W=0.235, df=3, P=0.872; sex: F/W=16.530, df=1, ***P<0.001; Blood Neutrophils: genotype: F/W=2.803, df=1, P=0.097; treatment: F/W=3.759, df=*P=0.013; sex: F/W=11.071, df=1, **P=0.001; Blood Eosinophils: genotype: F/W=0.155, df=1, P=0.694; treatment: F/W=10.871, df=3, ***P<0.001; sex: F/W=1.985, df=1, P=0.162; Blood Basophils: genotype: F/W=1.597, df=1, P=0.209; treatment: F/W=8.457, df=3,***P<0.001; sex: F/W=1.447, df=1, P=0.232;

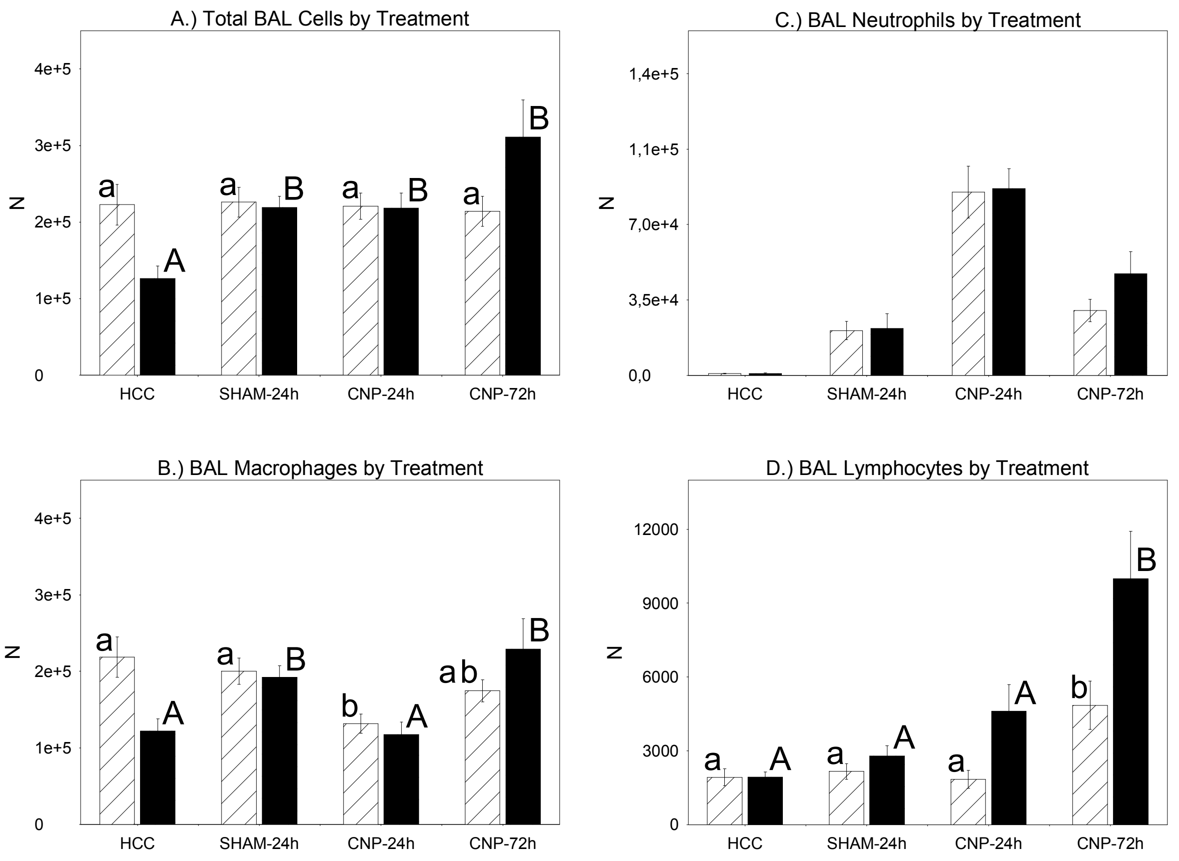

Additional Figure s4 - BAL cell differentials of male (white/coarse bars) and female mice (black bars) irrespective of genotype under different treatment conditions: untreated home cage controls (HCC; males/females: n=16/17); water-instilled animals at 24 h time point (SHAM-24h; males/females: n=14/15); particle-instilled mice at 24 h (CNP-24h; males/females: n=18/15) and 72 h time point (CNP-72h; males/females: n=16/13).

*Statistics: Post-hoc Tukey test after General Linear Model (GLM): different letters indicate significant differences between treatments within one sex at least at *P<0.05.*
